# Spatio-Temporal Synchrony of Influenza in Cities across Israel: The “Israel Is One City” Hypothesis

**DOI:** 10.1371/journal.pone.0091909

**Published:** 2014-03-12

**Authors:** Oren Barnea, Amit Huppert, Guy Katriel, Lewi Stone

**Affiliations:** 1 Biomathematics Unit, Department of Zoology, Faculty of Life Sciences, Tel-Aviv University, Tel-Aviv, Israel; 2 The Gertner Institute, Chaim Sheba Medical Center, Tel Hashomer, Israel; 3 Department of Mathematics, ORT Braude College, Karmiel, Israel; 4 School of Mathematics and Geospatial Sciences, RMIT University, Melbourne, Victoria, Australia; Duke-NUS Gradute Medical School, Singapore

## Abstract

We analysed an 11-year dataset (1998–2009) of Influenza-Like Illness (ILI) that was based on surveillance of ^∽^23% of Israel's population. We examined whether the level of synchrony of ILI epidemics in Israel's 12 largest cities is high enough to view Israel as a single epidemiological unit. Two methods were developed to assess the synchrony: (1) City-specific attack rates were fitted to a simple model in order to estimate the temporal differences in attack rates and spatial differences in reporting rates of ILI. The model showed good fit to the data (R^2^  =  0.76) and revealed considerable differences in reporting rates of ILI in different cities (up to a factor of 2.2). (2) A statistical test was developed to examine the null hypothesis (H_0_) that ILI incidence curves in two cities are essentially identical, and was tested using ILI data. Upon examining all possible pairs of incidence curves, 77.4% of pairs were found not to be different (H_0_ was not rejected). It was concluded that all cities generally have the same attack rate and follow the same epidemic curve each season, although the attack rate changes from season to season, providing strong support for the “Israel is one city” hypothesis. The cities which were the most out of synchronization were Bnei Brak, Beersheba and Haifa, the latter two being geographically remote from all other cities in the dataset and the former geographically very close to several other cities but socially separate due to being populated almost exclusively by ultra-orthodox Jews. Further evidence of assortative mixing of the ultra-orthodox population can be found in the 2001–2002 season, when ultra-orthodox cities and neighborhoods showed distinctly different incidence curves compared to the general population.

## Introduction

Influenza epidemics have been a major challenge for health care systems around the world year in year out [1]. Although influenza illness is usually limited to several days of relatively minor symptoms, it is not uncommon that influenza leads to pneumonia, mostly among children and the elderly, which might lead to hospitalization and even death [2]. The annual number of influenza cases worldwide is estimated at ^∽^1 billion, with 3–5 million of these developing severe illness and 300,000–500,000 cases ending in death [3].

**Figure 1:**
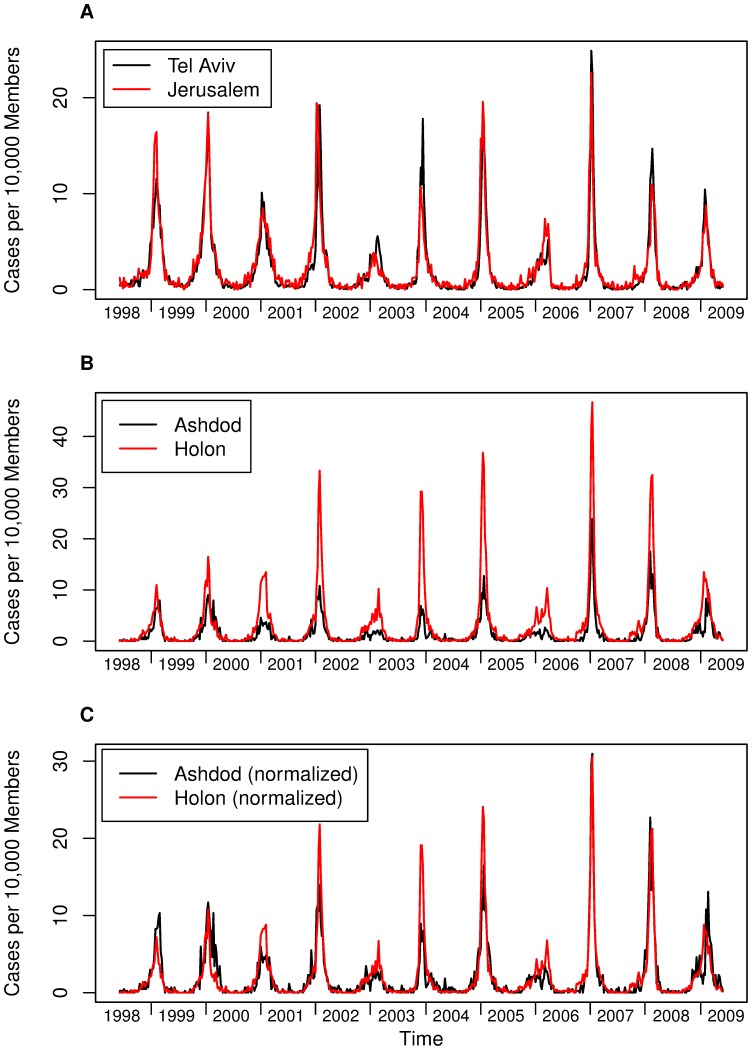
ILI incidence in several Israeli cities, 1998–2009. (A) Weekly number of ILI cases per 10,000 Maccabi Health Services members in Tel Aviv and Jerusalem the two largest cities in Israel. Note the high spatio-temporal synchrony. (B) Weekly ILI incidence in Holon and Ashdod. Incidence rates in Holon are generally much higher than Ashdod's. (C) same dataset as in B after normalizing each city's incidence by its calculated reporting rate using the Attack Rate/Reporting rate linear model method results (Table 1). Note that after incorporating the relative reporting rates the incidence curves look much more similar.

In the temperate regions influenza is predominantly seasonal, with epidemics occurring every winter, but the severity of the epidemic and the exact timing of the outbreak vary substantially between years. Figure 1A, for example shows these dynamical features for seasonal influenza time-series collected in Israel. The spatio-temporal characteristics of influenza epidemics including synchrony have been studied at different geographical scales from the global scale [4], [5] through the scales of continents and large countries such as Europe, the US and Brazil [6]–[8] to midsize countries like Italy, Japan and France and smaller countries as Israel [9]–[12]. These studies identified different spatial patterns including waves of influenza incidence [6]–[8], [10] and varying levels of synchrony, depending, to a degree, on the geographical scale [4], [5], [7], [9]. Analyses of this nature are important to understand how influenza spreads on a broad spectrum of geographical scale.

We propose to study the synchrony of influenza in a range of Israeli cities based on a high quality long-term database that has become available from Israel's Maccabi Health Service providers [12], [13]. Figure 1A shows the weekly incidence of influenza-like illness (ILI) between the years 1998–2009 in Israel's two main cities, Tel Aviv and Jerusalem. The epidemics in both cities exhibit an extraordinary degree of similarity both in their timing and their magnitude, as shown in previous work on influenza epidemics in Israel [12]. Tight synchrony between different cities raises the question as to whether Israel is small enough to be considered as a single epidemiological unit where influenza is concerned – we refer to this as the “Israel is One City” hypothesis. This possibility is supported by Israel's small size and the fact that over 40% of its population lives in the Dan metropolitan area. However, there are cases in which pronounced differences can be seen in the timing and/or magnitude of influenza epidemics in different cities in Israel. This can be attributed to real differences between the epidemics in Israel's cities, to weaknesses in the data, or variation in the rate of reporting of ILI in different cities. The latter feature was noted by London and Yorke [14] in their study of measles, chickenpox and mumps data in the US. To further improve our understanding of the spatial spread of influenza we examine the “Israel is One City” hypothesis more formally using two different approaches. First, we present a statistical method to investigate whether the attack rates of ILI over an epidemic outbreak are the same for all cities in each season, up to a scaling factor of the reporting rate. Second, based on daily incidence data, we present a statistical method that compares the epidemic curves of two cities and determines whether or not they are essentially identical over a single season.

### Data

This work is based on an ILI dataset supplied to by Maccabi Health Services (MHS), Israel's second-largest health maintenance organization which serves close to a quarter of Israel's population. (During the period of the research Maccabi's market share increased from 20.8% in 1998 to 24.8% in 2009.) The dataset includes all ILI cases diagnosed in Israel by Maccabi's doctors every day between January 1^st^ 1998 and May 31^st^ 2009; a total of 11 influenza seasons. The doctors all made use of ILI ICD9 diagnosis code 487.1 for influenza, as well as several MHS internal codes for influenza and influenza-like disease. In an analysis that appears elsewhere, we have confirmed the relationship between this ILI data and true influenza cases as based on serological data ([15], Appendix A). The dataset includes information about the patients' approximate place of residence. Cases of repeat ILI diagnoses (i.e., ILI diagnoses which occurred less than 28 days after a previous ILI diagnosis) for the same person, were excluded from the dataset.

Most influenza seasons in this period were dominated by the influenza A H3N2 virus, with the exceptions of 2000-1 and 2007-8, which were dominated by a H1N1 strain, 2002-3 and 2005-6 when influenza B was dominant, and 2008-9 which had no dominant strain (see table 1). Influenza A H3N2 seasons were characterized by higher attack rates compared to other seasons [12].

**Table 1:** Estimates of the RR/AR model for the attack rate of each season relative to the attack rate of season 1.

Season	Attack rate (95% CI)
1	1
2	1.19 (0.98 1.46)
3 (H1N1)	0.83 (0.69 1.01)
4	1.16 (0.95 1.41)
5 (B)	0.46 (0.38 0.56)
6	0.80 (0.66 0.97)
7	1.02 (0.84 1.25)
8 (B)	0.49 (0.40 0.59)
9	1.07 (0.88 1.31)
10 (H1N1)	0.92 (0.75 1.12)
11 (NDS)	0.54 (0.44 0.66)

A large fraction of registered ILI cases are in fact not influenza but other diseases with similar symptoms [16], [17]. The fraction of true influenza cases out of the ILI cases changes seasonally, since during the summer most cases of ILI incidence are not cases of influenza, while in the winter the fraction of true influenza cases is much higher [18]. In order to obtain a better estimate of influenza cases, ILI cases from before and after the influenza season were eliminated. The Israeli Center for Disease Control sends samples from sentinel clinics to laboratory tests for identification of influenza strains, and the tests show that the length of influenza season is between 14 and 19 weeks (data is available in Hebrew weekly reports similar to [18] and on WHO's FluNet website). Therefore, a period of 120 days (^∽^17 weeks) in each winter containing the highest number of ILI cases, most of which are influenza cases, was used to estimate the attack rate of influenza in each season. Other definitions for an ILI season length, ranging from 90 days to 180 days, were examined as well and gave similar results.

**Figure 2:**
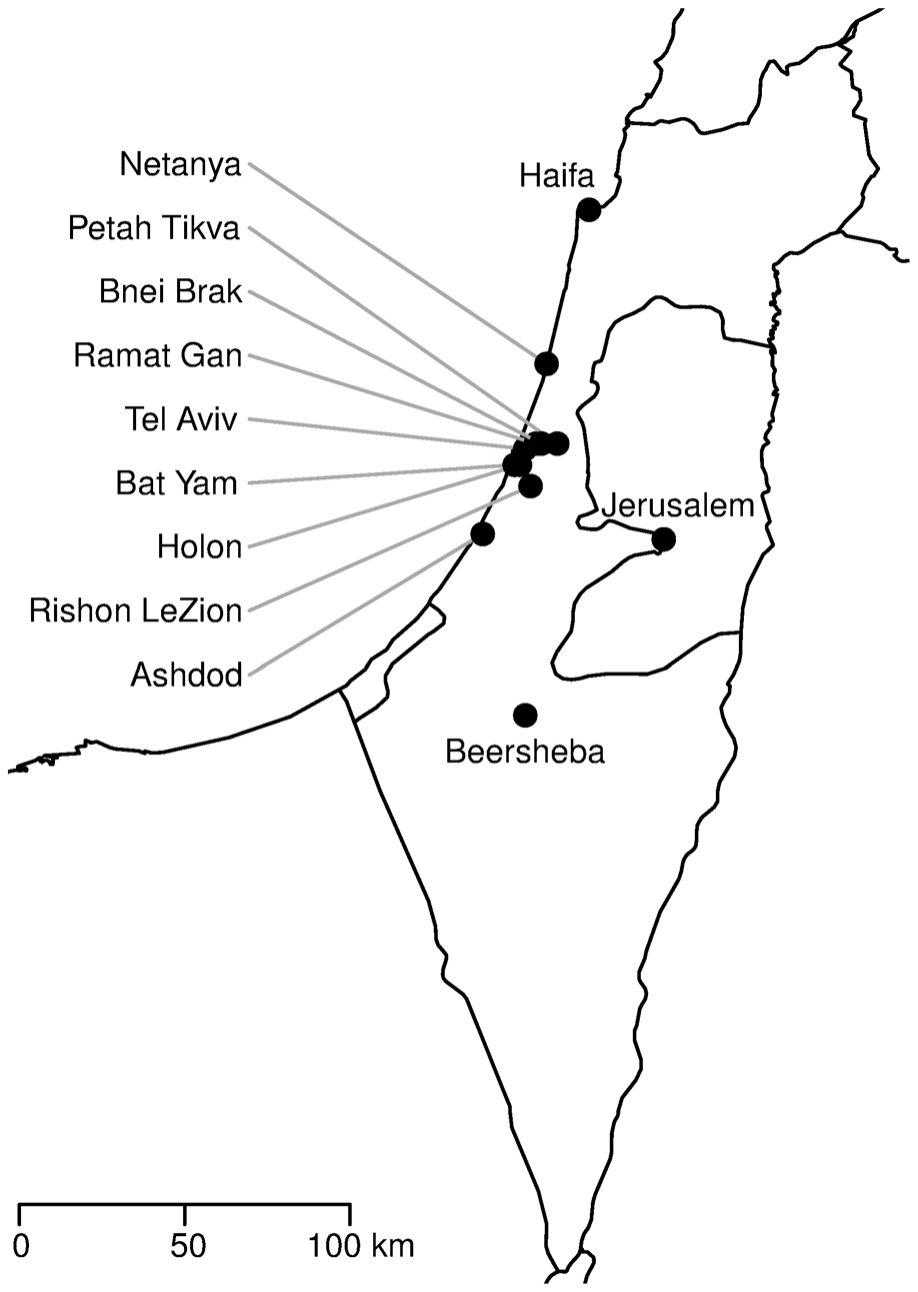
A map of Israel and the 12 cities analysed in this work.

Twelve cities were studied in this work, each having over 30,000 MHS members in 1998. Smaller cities were found unreliable because it was found that cities with few doctors could have large classification biases that introduced major surveillance errors. Figure 2 shows the location of the twelve cities in Israel. With the exception of the far north and far south, this set of 12 cities covers most of Israel's area. Seven of the 12 cities are located in the Dan Metropolitan Area: Tel Aviv, Rishon LeZion, Bnei Brak, Holon, Bat Yam, Ramat Gan and Petah Tikva. Three other cities are located on the Israeli coastal plain, with Haifa and Netanya being north of Tel Aviv (^∽^80 km and ^∽^25 km, respectively) and Ashdod being ^∽^33 km south of Tel Aviv. The remaining two cities are Jerusalem, ^∽^50 km south-east of Tel Aviv and Beersheba, ^∽^90 km south of Tel Aviv.

## Results

### 1. Reporting Rate and Attack Rate Model (RR/AR)

Figure 1B displays the weekly ILI incidence curves of two cities in Israel, Ashdod and Holon (34 km apart), over 11 years (1998–2009). The two cities show a large degree of synchrony, but the ILI incidence in Holon is consistently higher than in Ashdod. However, upon multiplying the incidence curves by an appropriate scale factor, the two cities have almost identical dynamics in time (Figure 1C). The synchrony thus revealed is very similar to that we have already seen for the time series of Jerusalem and Tel Aviv in Figure 1A. This same manifestation of synchrony was found to be widespread between most pairs of cities after appropriate rescaling. There were, however, cases where the property was unclear. Motivated by these interesting findings, we were led to ask to what degree ILI epidemics in any pair of cities is actually the same for each season, but the rate of reporting ILI cases might be different in each city.

In the first test, rather than study the daily time series of each city over the 11 year period, we restrict our study to the observed seasonal attack rates. The attack rate of a single epidemic is defined here as the proportion of people diagnosed with ILI during a 120 day season out of the total number of Maccabi members in that city in that season.

The reporting rate-attack rate (RR/AR) model rests on two assumptions:

- each influenza season (*s*) has the same attack rate *a*_s_ (*s*  =  1…11) for all cities. The justification behind this assumption relates to Figure 1 where we visually see this holding for the two major cities, Tel Aviv and Jerusalem;
- that each city (*c*) has its particular reporting rate *r*_c_ (*c*  =  1…12) which is constant throughout the 11-season period of the dataset.

The observed attack rate *i*_c,s_ in city *c* over season *s* is the product of these two values *a*_c_ *r*_s_. Any deviation from this product is regarded as an independent random effect. The RR/AR model may therefore be written as
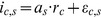
(1)


where *ε*_c,s_ is residual stochastic noise having mean zero. The Methods section details the model fitting technique. There we estimate the best fitting attack rate parameters *a*_s_ and the reporting rate parameters *r*_c_ from the observed attack rates *i*_c,s_.

Note that an infinite number of sets of parameters *a*_1…11_ and *r*_1…12_ can give an optimal fit to the data, since it is possible to multiply all attack rates *a*_s_ by a constant and divide all values of *r*_c_ by the same constant to obtain the exact same fit. However, the ratios between the cities' reporting rates will remain the same, as will the ratios between the seasons' attack rates. These ratios of attack rates are of interest since they give indication of the differences in the epidemiological properties of the dominant strain of influenza in each year (such as infectiousness and prior susceptibility) and the ratios of reporting rates give indication of each city's reporting properties (e.g. the tendency of doctors to diagnose diseases as ILI).

Table 1 and Table 2 present the attack rates (*a*_s_) and reporting rates (*r*_c_) respectively as found through fitting the model to the observed data *i*_c,s_ for the twelve cities in Israel over the years 1998–2009. The attack rates are all relative to the first season (in 1998–1999) having taken *a*_1_  =  1 and the reporting rates are relative to Tel Aviv where *r*_tel aviv_  =  1.

**Table 2:** Estimates for the reporting rate of each city relative to the reporting rate of Tel Aviv (Relative Reporting Rate, RRR) found using the RR/AR model (middle) and the incidence curves comparison method (right).

City	RRR, RR/AR model (95% CI)	RRR, ICC method
Ramat Gan	1.69 (1.38 2.08)	1.68
Holon	1.53 (1.25 1.88)	1.53
Rishon LeZion	1.42 (1.17 1.73)	1.41
Petah Tikva	1.36 (1.11 1.66)	1.35
Bnei Brak	1.36 (1.12 1.65)	1.36
Bat Yam	1.15 (0.94 1.42)	1.15
Jerusalem	1.05 (0.86 1.28)	1.05
Beersheba	1.04 (0.85 1.28)	1.04
Tel Aviv	1	1
Haifa	0.95 (0.77 1.17)	0.95
Ashdod	0.77 (0.63 0.94)	0.79
Netanya	0.80 (0.66 0.97)	0.76

**Figure 3:**
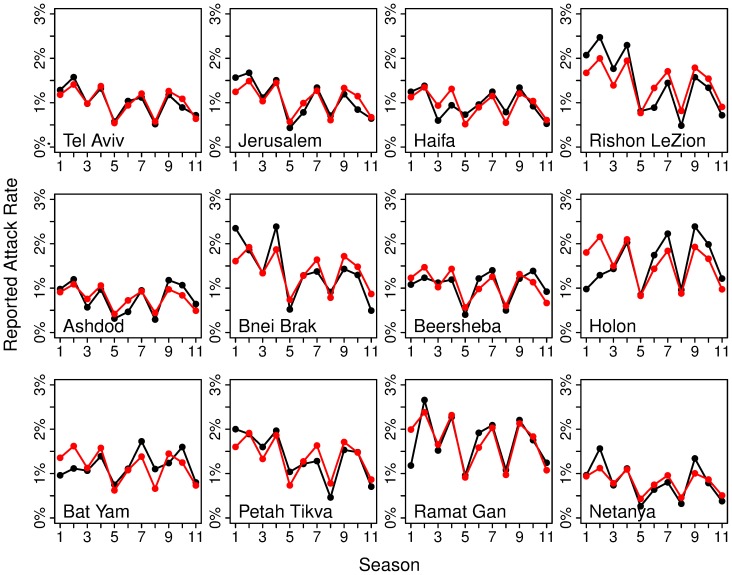
Observed vs. expected attack rates. Reported attack rates in each city in each season (black) compared to expected results from model fits (red) using equations 5–6. The expected attack rates in all cities are identical up to a scaling factor of the reporting rate. Note the good fit between the expected and observed attack rates in most cities in most seasons.

As shown in the Methods section, the above model explains a surprisingly large 75.5% of the variance in the data indicating that the estimates *a*_s_ and *r*_c_ fit the data well, and corroborating the validity of the model. Figure 3 shows the fits in more detail by comparing the observed *i*_c,s_ and the expected city-specific attack rate (*a*_s_ *r*_c_) in each city for each season. Note that the expected attack rate of each season is identical in all cities up to a scaling factor, which is the reporting rate typical for each city, and the differences in reporting rates of different cities are very pronounced (see table 2).

While the attack rates table (table 1) can be estimated directly from the observed attack rates of all of Israel in each season (and indeed the two sets of results are very well correlated, r^2^  =  0.96, p  =  1.5 · 10^−7^), the table of estimated relative reporting rates (table 2) supplies information which cannot be obtained directly from the data, and might be of considerable importance for disease management. The high reporting rates in Ramat Gan and Holon, for example, might indicate that the doctors there have a higher tendency to diagnose a disease as influenza compared to doctors in other places, a problem which was discussed before [12], [19].

Figure 1C shows the weekly incidence of ILI in Holon and Ashdod after normalization for reporting rates obtained from Table 1. The two curves are clearly more similar than their raw (i.e., unnormalized) counterpart curves in figure 1B.

### 2. Comparison of Incidence Curves

**Figure 4:**
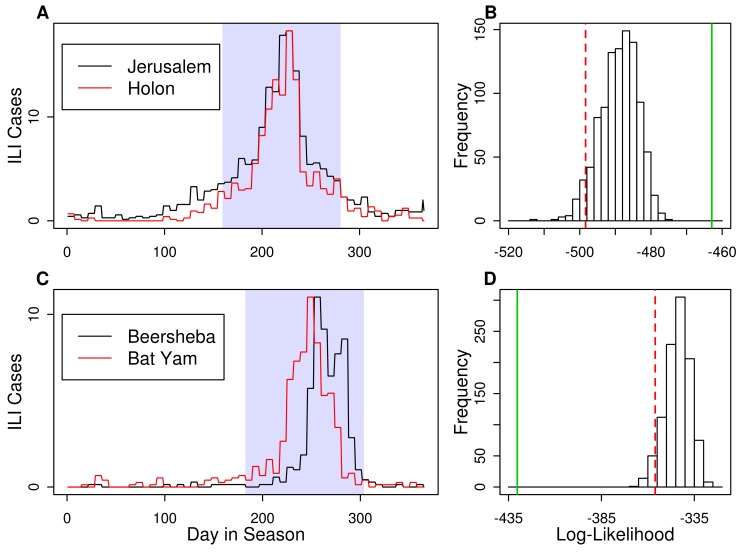
Comparison of Incidence Curves: two examples. **Top (A,B):** Jerusalem and Holon in the 1999–2000 season. Incidence time series curves (A) are very similar. A period of 120 days containing the largest number of ILI cases (highlighted with a darker band) was used to calculate the likelihood of the observed incidence curves. Histogram of log-likelihoods (B) of 1,000 simulated epidemics (equations 20–22) gives a one-sided 95% confidence limit (dashed red line) and the log-likelihood of the real data (solid green line) is very high compared to those of simulated epidemics, indicating that simulated epidemics are less similar than the observed data. **Bottom (C,D):** Beersheba and Bat Yam in the 2008–2009 season. Dramatic differences in the shape of the incidence curves (C) translate to a very low log-likelihood of the observed epidemic compared to those of simulated ones (D).

In the previous section we tested and compared seasonal attack rates of all cities. Here we investigate the "Israel is One City" hypothesis with a more fine temporal scale analysis by checking whether two individual epidemic outbreak curves are statistically identical over a given period based on incidence data analysed on a weekly time-scale. For example, Figure 4A shows the epidemic outbreaks for Holon and Jerusalem in the same season 1999–2000, and the two curves appear almost identical by eye. In Figure 4B, however, the epidemic curves of Beersheba and Bat Yam for the 2008–2009 season appear to be significantly different: Bat Yam's ILI incidence rises above baseline level ^∽^25 days before Beersheba's incidence and peaks earlier. Towards the end of the season, ILI incidence in Beersheba remains very high when Bat Yam is already at baseline level.

In the Methods section, a statistical test is presented for identifying whether the epidemic curves associated with two cities are identical up to a scaling factor (reporting rate), with differences between cities only due to random sampling effects. More formally, by ‘identical epidemic' we mean that individuals in both cities have the same chance of getting infected for any specific day *t*. This leads us to define the infection probabilities *p_t_* (1 ≤ *t* ≤ *T*) as the probability that any person (in any city) becomes infected on day *t*. The null hypothesis (H_0_) that we wish to test is therefore that both cities share the same probabilities of infection *p_t_*.

The method calculates the expected number of ILI cases in each day of the epidemic in each city, based on the total number of ILI cases that day in both cities. If the population numbers of city *k* is *N*_k_, then the expected value for the number of new ILI cases on day *t* in city *k* is
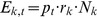
(2)


Here the reporting rate *r_k_* for city k (1 ≤ *k* ≤ *n*) is the probability that a person who becomes infected goes to the doctor, and is diagnosed with influenza.

Based on a maximum likelihood approach, the method is able to estimate the *T* parameters *p_t_* and the two reporting rates *r*_1_ and *r*_2_ from the ILI data for each of two cities. Once the *E*_k,t_ are estimated it then becomes possible to simulate epidemics under the null hypothesis that both cities share the same probabilities of infection *p*_t_. The simulation of the epidemic is driven by a Poisson model
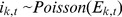
(3)


where *i*_k,t_ is the number of newly infected individuals on day *t* in city *k*. The null hypothesis is tested by comparing the likelihood of the simulated epidemics to the likelihood of the observed data.

The procedure for testing the null hypothesis is outlined in the following steps for a given pair of cities, say cities 1 and 2:

- The parameters *p_t_* and *r_k_* are estimated from the ILI data of the epidemic curves of the two cities (see Methods) for *k*  =  1,2 and *t*  =  1,2,…,*T*.
- One thousand simulated epidemics *i*_k,t_ for both cities are generated (*k*  =  1,2; *t*  =  1,2,…*T*) from Equation 3.
- The histogram of the log-likelihoods (Equation 21) of city pairs is determined, as shown in Figure 4.
- One-sided 95% confidence limits (dashed red line in Figure 4) are calculated for the log-likelihood distribution.
- The null hypothesis is rejected if the log-likelihood of the observed data falls outside the 95% confidence limit.

The log-likelihood histograms in Figure 4 illustrate the test in practice. The null hypothesis (H_0_) that the epidemic curves of Jerusalem and Holon are identical cannot be rejected since the observed log-likelihood falls inside the 95% confidence limits. In contrast the observed log-likelihood of the cities of Beersheba and Bat Yam (2008-2009) falls outside the 95% confidence limits and the null hypothesis must be rejected.

It is possible to analyse all city pairs in this fashion. For the 12 Israeli cities, there are 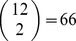 different pairs of cities over 11 influenza seasons, giving a total of 726 combinations of pairs of cities in different seasons. Of these combinations, the null hypothesis (H_0_) could not be rejected in 77.4% of the cases. The fraction of city pairs with different incidence curves (i.e. pairs for which H_0_ was rejected) in each season varied between 30 different pairs in the 2008-2009 season (45.5% of all pairs in the season) and 5 different pairs in the 2002-2003 season (7.6%).

Considerable variance is also seen in the fraction of different combinations which include specific cities (table 3). Netanya was found to have a different incidence curve to the other city in just 7 out of 121 combinations (5.8%), followed by Jerusalem which had different incidence curves in 14% of the combinations and Rishon LeZion with 15.7%. The cities with the highest fraction of differences were Bnei Brak with 43.8%, Beersheba with 37.2% and Haifa with 33.9%. It can be argued that these three cities are the three most disconnected cities in the 12-city set used here (see Figure 2), since Beersheba and Haifa are geographically the furthest cities from the Dan metropolitan area and Bnei Brak (an ultra-orthodox city) is socially the most disconnected from most other cities (see also Discussion).

**Table 3:** Fraction of combinations (two cities and a season) in which incidence curves were found to be significantly different, out of each city's 121 combinations.

City	% Different Combinations
Bnei Brak	43.8%
Beersheba	37.2%
Haifa	33.9%
Ramat Gan	28.1%
Petah Tikva	23.1%
Bat Yam	18.2%
Ashdod	17.4%
Holon	17.4%
Tel Aviv	16.5%
Rishon LeZion	15.7%
Jerusalem	14.0%
Netanya	5.8%

Since this method estimates the reporting rate of ILI in each of the two cities in a given season, we averaged all 121 estimated reporting rates of each city and compared them to the average reporting rate of Tel Aviv, in order to confirm the results of the RR/AR method. Table 2 shows that the results of both methods are very similar.

A correlation matrix of daily incidence rates was calculated as an additional index of synchrony between all pairs of cities (table 4). The average correlation between each pair of cities was 0.766. The average correlation of each city with other cities was calculated and most values were close to the mean, ranging between 0.692 (Bnei Brak) and 0.828 (Tel Aviv). These high correlations are to be expected in light of our analysis of the epidemic curves between cities. The lowest correlations are associated with Bnei Brak which was found to have the lowest degree of synchrony by the epidemic curve comparison method as well.

**Table 4:** Correlation matrix of daily incidence rates in all city pairs in 1998–2009.

City	TLV	JER	HFA	RSN	ASD	BBK	BSV	HLN	BYM	PTV	RMG	NYA	Mean
Tel Aviv	1.00	0.83	0.85	0.85	0.77	0.74	0.79	0.86	0.80	0.82	0.80	0.84	0.83
Jerusalem	0.83	1.00	0.78	0.76	0.69	0.70	0.74	0.77	0.74	0.73	0.75	0.74	0.77
Haifa	0.85	0.78	1.00	0.80	0.71	0.68	0.76	0.84	0.77	0.75	0.76	0.77	0.79
Rishon LeZion	0.85	0.76	0.80	1.00	0.71	0.74	0.73	0.81	0.77	0.79	0.74	0.78	0.79
Ashdod	0.77	0.69	0.71	0.71	1.00	0.61	0.69	0.77	0.70	0.72	0.68	0.73	0.73
Bnei Brak	0.74	0.70	0.68	0.74	0.61	1.00	0.59	0.63	0.65	0.68	0.65	0.62	0.69
Beersheba	0.79	0.74	0.76	0.73	0.69	0.59	1.00	0.81	0.73	0.70	0.72	0.73	0.75
Holon	0.86	0.77	0.84	0.81	0.77	0.63	0.81	1.00	0.83	0.78	0.78	0.78	0.81
Bat Yam	0.80	0.74	0.77	0.77	0.70	0.65	0.73	0.83	1.00	0.71	0.75	0.70	0.76
Petah Tikva	0.82	0.73	0.75	0.79	0.72	0.68	0.70	0.78	0.71	1.00	0.70	0.74	0.76
Ramat Gan	0.80	0.75	0.76	0.74	0.68	0.65	0.72	0.78	0.75	0.70	1.00	0.72	0.75
Netanya	0.84	0.74	0.77	0.78	0.73	0.62	0.73	0.78	0.70	0.74	0.72	1.00	0.76

### 3. Ultra-Orthodox Population

**Figure 5:**
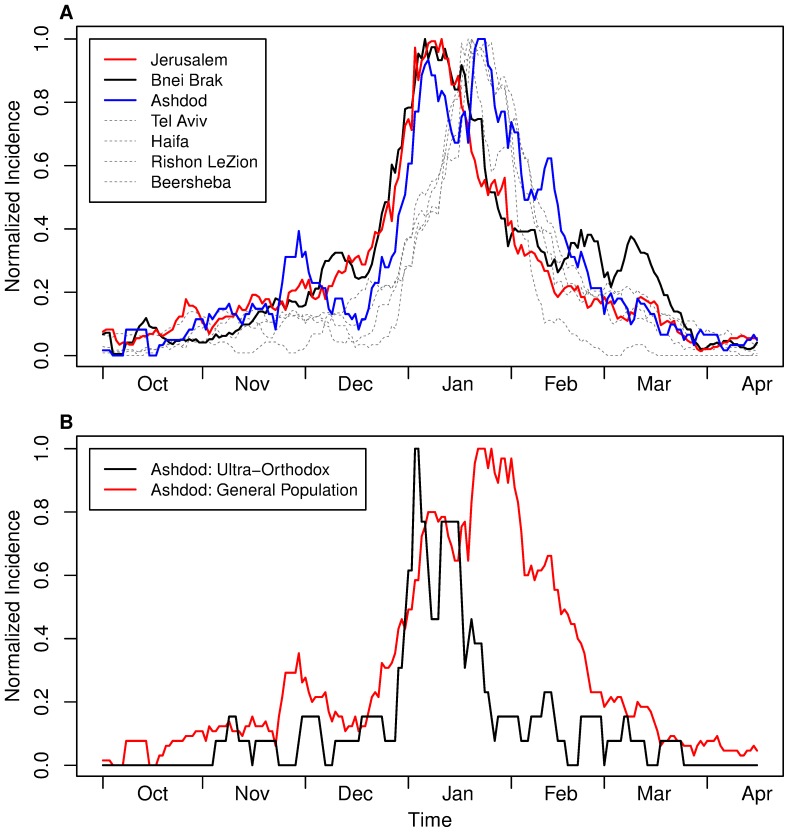
ILI incidence in the ultra-orthodox population during the 2001–2002 season. (A) ILI incidence in Jerusalem (red), Bnei Brak (black), Ashdod (blue) and Israel's main cities – Tel Aviv, Haifa, Rishon LeZion and Beersheba (dashed gray). Incidence curves were normalized by dividing each incidence curve to its own maximum in order to equalize all maxima and emphasize the degree of synchrony. Note early January peak in Jerusalem and Bnei Brak, a later January peak in all other cities, and a double peak in Ashdod. (B) ILI incidence in same season in the two different population groups of Ashdod: Ultra-Orthodox (black) and the general population (red). The General population of Ashdod shows a similar incidence curve to other non-Orthodox cities, while the ultra-orthodox parts of the city resemble the cities which large ultra-orthodox communities.

An interesting phenomenon of a distinctly different influenza epidemic in a sub-population of Israel, as compared to that experienced by the rest of the country, can be seen in the 2001–2002 season. The epidemics of most cities in Israel were well-synchronized in this season and peaked around January 20th, except Jerusalem and Bnei Brak. The latter cities peaked earlier in January 11th and January 6th respectively and had very similar incidence curves throughout the season. Even more unusually, Ashdod had a double-peak incidence curve in that season, with the first peak occurring on January 8th and the second peak on January 22nd (Figure 5A). We find that these early epidemics could well arise from the impact of significant religious ultra-orthodox communities in these cities.

Jerusalem and Bnei Brak have very dominant ultra-orthodox communities (e.g. in 2012 all but one elementary schools in Bnei Brak are ultra-orthodox schools, and ^∽^62% of the Jewish schoolchildren in Jerusalem attend ultra-orthodox schools [20]). Since Ashdod has a significant ultra-orthodox population, the data from Ashdod can be divided into "mainly ultra-orthodox " areas and "general secular population" areas, based on an Israeli Central Bureau of Statistics report [21]. A separate incidence curve was thus created for each population type. It appears that the secular population of Ashdod is well synchronized with the overall population of Israel, while the incidence curve of the ultra-orthodox population of Ashdod shows remarkable resemblance to those of Jerusalem and Bnei Brak (Figure 5B). This might be an indication for a separate outbreak of ILI in the ultra-orthodox population, a phenomenon which was shown before in other diseases such as measles [22] and mumps [23].

This result is supported by comparisons of the incidence curves of pairs of cities in the 2001–2002 season. The pairwise incidence curves of Jerusalem, Bnei Brak and Ashdod were found not to be different. When compared to the other 9 cities in the dataset, Bnei Brak's incidence curve was found to be different to all other cities in the dataset except Netanya. Jerusalem's incidence curve was found to be different to those of all cities except Netanya, Ramat Gan and Petah Tikva. Ashdod was found not to have a different incidence curve to any other city. Comparing the incidence plots of the ultra-orthodox population and the general population in other cities was not possible due to the lack of data regarding the number of Maccabi members in each population group.

## Discussion

In this work we explore the degree of spatio-temporal synchrony of influenza in Israel using a high quality ILI dataset (see also [12]). Perfect synchrony of ILI epidemics would be manifested in identical attack rates and identically-shaped incidence curves in all cities. Examination of both of these properties indicates that to a surprisingly high degree, Israel's ILI incidence can be seen almost as a single, homogenous epidemic in all 12 cities analysed in this work.

It was previously shown that some degree of synchrony of influenza epidemics exists in all spatial scales up to the scale of a hemisphere [4], [5], [11]. It appears, however, that distance reduces the level of local synchrony. Closer US states show more correlation in influenza timing and amplitude than states which are further apart [7]. In addition it was previously shown that the level of synchrony between Israel and France is high, nevertheless it is lower than the level of synchrony between Tel Aviv and Jerusalem which are 62 km apart [12]. Here we extend the study to include 12 different cities to further investigate the degree of synchrony of ILI epidemics in Israel. Israel's small size (^∽^22,000 km^2^) combined with a high degree of population mixing potentially lead to a high degree of synchrony, possibly to an extent to which it can be regarded as a “single city”. Additionally, the fact that ^∽^40% of its population is concentrated in the Dan Metropolitan Area, a small and dense region in central Israel (^∽^1,500 km^2^), further decreases the “effective area” of Israel which should intensify the population mixing and affect the epidemic synchronization between the cities in this area, which contains 7 out of the 12 cities analysed in this work. Unlike childhood infectious diseases (e.g. measles) in which transmission is localized due to the contact patterns of children usually limited to their schools, neighborhoods and close families [7], influenza is a disease transmitted between individuals of all ages, which could be a significant factor contributing to highly-synchronized epidemics.

On the other hand, some geographical, environmental and social properties of Israel contribute to the de-synchronization. Despite its small size, climate conditions in different areas of Israel are quite different, in both temperature and humidity, both of which are known to affect influenza transmission [15], [24]–[27]. These differences in climate conditions are potentially substantial enough to cause differences in the timing or magnitude of influenza epidemics in different cities. There is also a considerable degree of social heterogeneity in the Israeli population, e.g. regions with concentrations of minority populations such as Arabs and ultra-orthodox Jews. These sub-populations of the Israeli society have distinctive social, demographic and economic characteristics which are known to have effects on their mixing with the more general population. For instance, during the pandemic in Israel it was shown that the disease spread faster in soldiers and the orthodox community [28].

In order to obtain a measure of the differences in the severity of influenza epidemics in different cities we examined the variation in ILI reporting rates in different cities using a simple linear model (the RR/AR method), which explained 75.5% of the variance in the data. The RR/AR model examines the assumption that attack rates in all cities in a given season are the same. The model has a built-in parallel assumption of constant, typical reporting rate in each city that may differ between cities. Had these two assumptions been absolutely correct, the model would explain 100% of the variance in the data. Although the result of 75.5% is remarkably high, it also indicates that these assumptions are not fully realized in practice and additional causes for differences in attack rates, which are not incorporated in this model, might also exist.

The assumption of common attack rate for each season might be justified on the basis that with the exception of one season, each influenza season is characterized by a single dominant influenza strain [12], [15]. Although it is known that on a larger geographical scale there are local outbreaks of different subtypes of influenza [29], no data exists for the exact dominant subtype of influenza in each city in Israel. Since in 10 out of 11 seasons in the dataset there was a single dominant subtype and the geographical spread of the different subtypes in the remaining season is unknown, the assumption of spatial homogeneity of influenza strains seems reasonable. We have also assumed that the reporting rates of ILI vary between different cities but are constant in time. The reporting rates might differ between cities for at least two reasons: population-related and influenza diagnosis related. Population-related reasons include socio-demographic properties of cities, such as age structure, household size and unemployment rates, which can all affect the patients' tendency to seek doctor consultation when they have influenza symptoms. We assume, for example, that unemployed people are less likely to visit a doctor when they have ILI symptoms since they do not need illness certificates. In order to test this assumption we checked the correlation between the estimated reporting rate and the employment rate of each city. Employment data is published annually and is available for each year since 2002 [30]. Each city's mean employment rate in 2002–2009 was correlated against each city's reporting rate and was found to be positive and significant (p  =  0.031, R^2^  =  0.38). This finding is in agreement with those of Charland et al (2011) who examined the relationship between rates of influenza and level of material and social deprivation [31].

Influenza diagnosis affects the variance in reporting rates since different doctors have different tendencies to diagnose a disease as ILI. Influenza shares most of its symptoms with other diseases, such as common cold [16] RSV and parainfluenza [17]. It is therefore possible that, especially in the smaller cities, a small number of doctors who tend or don't tend to diagnose diseases as ILI may lead to a higher reporting rate of ILI, or vice versa (this is known as a classification bias). These properties are assumed to change at a slow rate, and therefore each city's reporting rate is assumed to be relatively constant over the 11-season period. Our model shows that the differences between reporting rates of different cities are quite dramatic, with a factor of 2.2 between the highest and lowest reporting rates (see table 2). Although this factor might seem large, it is actually similar to what London and Yorke [14] noted for measles in the US.

The “shapes” of ILI incidence curves of all pairs of Israel's 12 largest cities in each season were compared using a likelihood-based test. The test was applied on 726 combinations of two cities and a season, and found that in 77.4% of the combinations the incidence curves are not distinctly different, again indicating a high degree of spatial and temporal synchrony between the cities. Additionally, this method shows that while some cities are well-synced with other cities, a few typical cities show relatively poor synchrony. Each city appeared in 121 pairs out of the total of 726 pairs, and some cities had more pairs that were significantly different in terms of their incidence curves than other cities (i.e., “out of sync” incidence curves). The cities with the largest fraction of “out of sync” curves are Beersheba, the furthest large city from the Gush Dan area located in the Negev desert (i.e., different climate conditions and remoteness), and Bnei Brak, a city in the heart of Gush Dan populated almost exclusively by ultra-orthodox Jews (see table 3 for full results). The low synchrony of Bnei Brak compared to other cities might indicate that the coupling between Bnei Brak's population with the general population of Israel is lower than the coupling between the non-orthodox cities. Further evidence that assortative mixing can lead to unique dynamics can be found in the early outbreak of ILI in the ultra-orthodox communities in 2001–2002 (Figure 5). This outbreak is characterized by an “ultra-orthodox peak” which occurred ten days before the general population but synchronized between the “ultra-orthodox cities”. Nevertheless such dynamics are the exception rather than the rule, and were not observed in any of the other ten seasons.

Significantly different incidence curves between pairs of cities might be the result of random effects. For example, an influx of a few infectives in the early stages of the epidemic might be enough to change the shape of the incidence curve to a degree that makes it significantly different to the other city's curve. However, these cases seem to be uncommon. Excluding Bnei Brak and Beersheba, the fraction of significantly different combinations drops from 22.6% to 14.5%. When Haifa, the third-least synchronized city in the dataset (and the northernmost city in it) is excluded, the fraction drops to 10.6% of the combinations.

Although ILI incidence data for the 2009–2010 “swine flu” pandemic was available for this analysis, it was not included in it since this work focuses on seasonal influenza epidemics. However since a 120-day period is used here for both calculating attack rates and comparing incidence curves, the inclusion of the 2009–2010 season gives similar results. Israel had three waves of influenza during the 2009–2010 pandemic. The first two waves, which occurred in the summer and autumn, showed relatively low synchrony [28]. However the third wave, which occurred in the winter and had much higher incidence rates than the first two, had similar degree of synchrony to seasonal flu epidemics. With the 2009–2010 season included, the RR/AR method explained 71% of the variance in the attack rate data (compared to 75.5% in the 11-season dataset), the curves comparison method showed that 76.8% of the incidence curve pairs are not different (compared to 77.4%), and the average correlation between incidence curves of two cities was 0.773 (compared to 0.766).

Overall, it seems that as a first approximation,Israel's ILI epidemics are similar enough in both shape and severity to be considered as a “One City”. Subtle differences can be found in the incidence data, but these deviations from the “One City” hypothesis account for only ^∽^25% of the data in both the attack rates and incidence curves. These differences appear to arise for both geographical and social reasons.

## Methods

### 1. Reporting Rates and Attack Rate Model

Given the observed attack rate data *I*_c,s_ (*c*  =  1,2…,*n*, *s*  =  1,2…*m*) for *n* cities over *m* seasons, we show how to estimate the parameters *a*_s_ (*s*  =  1…*n*) and *r*_c_, (*c*  =  1…*m*) based on the model: 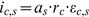
(4)


Since there is no single best-fitting set of parameters to this model (see below), we set the sum of all attack rates to unity, that is 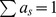. To find the typical reporting rate of each city, note:
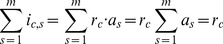
(5)


The typical attack rate of each season is found using 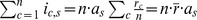


where 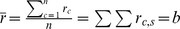, and thus
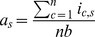
(6)


### 2. Comparing Incidence Curves of Two Cities

We present a bootstrap-based approach for testing the null hypothesis that incidence curves of two cities are identical, after taking into account that the cities may have different reporting rates. Given influenza incidence data for two cities over *T* days, with 1 ≤ *t* ≤ *T*, then our null hypothesis is that there is a vector *p*_t_ (1 ≤ *t* ≤ *T*) which gives the probability that any person, in any city, is diagnosed with ILI on day *t*. The constant reporting rate *r*_k_ for city *k* (*k*  =  1, 2) is the probability that an individual who becomes infected develops symptoms, goes to the doctor, is diagnosed with ILI, and that the case is reported.

Denote the population numbers of city *k* as *N*_k_. By the above, the expected value for the number of cases on day *t* in city *k* is
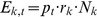
(7)


The probability that an individual in city *k* becomes infected and is reported is *p*_t_ *r*_k_, so that the number *i*_k,t_ of reported new cases is binomially distributed
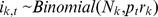



Since *N*_k_ is large, this distribution can be approximated by a Poisson distribution
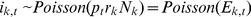
(8)


As shown shortly, the model parameters *p_t_* and *r*_k_ can be estimated from the data and the fit of the model to the data can be tested.

As in the attack rate model above, if we multiply all values *p*_t_ by a constant *c* and divide all the values *r*_k_ by the same constant, the model is left unchanged. So there is no possibility to identify the parameters uniquely. To overcome this indeterminacy, we set arbitrarily
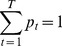
(9)


The likelihood function (the probability of obtaining the data *i*_k,t_, assuming model (2)), is given by:
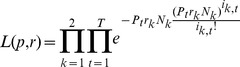
(10)


hence the log-likelihood is given by
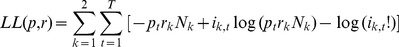
(11)


To fit the parameters we maximize LL with respect to *p*_t_, *r*_k_. Differentiating we have
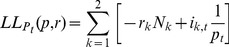
(12)

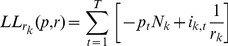
(13)


so the optimality conditions are
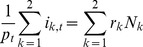
(14)

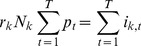
(15)


from (15) and (9) we have our maximum likelihood estimate for *^r^*_k_

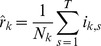
(16)


Plugging (16) into (14) we get our maximum likelihood estimate for *p*_t_

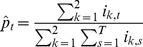
(17)


Note that 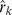 is simply the attack rate in city *k* (fraction of population who got infected during that season). 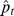 is the total number of reported cases (in both cities) on day *t*, divided by the total number of reported cases in the season. Because of the arbitrary normalization (4), these two parameters do not have their original meaning of reporting rate and probability of being infected, but as we noted above, this fact is of little importance, since we are only interested in the product 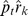, which retains its meaning as the probability that a person in city *k* on day *t* is infected and reported.

From (16) and (17) we get that the expected number of cases in city *k* on day *t* is
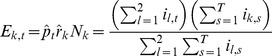
(18)


#### Testing goodness of fit

If our model is consistent with the data then we should have that i_k,t_ is ‘close’ to E_k,t_, for both cities k = 1,2, for all t. This was tested by generating 1,000 simulated epidemics for each one of the cities using the computed E_k,t_ values by generating 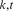 randomly according to
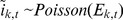
(19)


Then, the log-likelihood of each of the simulated epidemics was calculated:
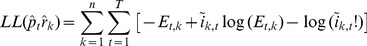
(20)


(note that the values *E*_k,t_ are computed using the real data, while 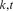 are obtained from the simulated data). 95% confidence intervals for 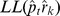 were obtained by finding the 50^th^ smallest value computed out of the 1,000 simulations, and comparing this value to the log-likelihood value computed for the real incidence data:
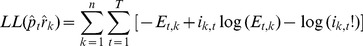
(21)


In other words, our H_0_ cannot be rejected if the log-likelihood of the real incidence data is not significantly smaller than the log-likelihood of the simulated epidemics. H_0_ is rejected if the log-likelihood of the real data is smaller than that of the 50^th^ smallest likelihood value of the simulated epidemics.

Since the raw data has a strong weekly cycle, with very few reported cases during the weekends, the data was summed over weeks and the incidence for each day was set to be 1/7^th^ of the total weekly incidence.
